# Management of a congenital tracheoesophageal fistula in a young Spanish water dog

**DOI:** 10.1186/1746-6148-10-16

**Published:** 2014-01-14

**Authors:** Pia S Kaminen, Sanna J Viitanen, Anu K Lappalainen, Anja Kipar, Minna M Rajamäki, Outi M Laitinen-Vapaavuori

**Affiliations:** 1 Department of Equine and Small Animal Medicine, Faculty of Veterinary Medicine, Helsinki University, Helsinki, Finland; 2 Veterinary Pathology, Department of Veterinary Biosciences, Faculty of Veterinary Medicine, Helsinki University, Helsinki, Finland

**Keywords:** Congenital tracheoesophageal fistula, Dog, Bronchoscopy, Fluoroscopy, Computed tomography, Histopathology

## Abstract

**Background:**

Tracheoesophageal fistula (TEF) in dogs is a rare disease with only few reports in the literature. This report aims to contribute to the current literature on suitable diagnostic methods for TEF and to provide follow-up information after successful surgical treatment.

**Case presentation:**

A seven-month-old intact female Spanish Water Dog was presented for further investigation of recurrent respiratory symptom. Bronchoscopy revealed a small hole-like defect in the tracheal wall at the bifurcation. The finding of the contrast material swallow study under fluoroscopy was indicative of a TEF. To further evaluate the connection between the trachea and esophagus, a computed tomography scan was performed. The TEF was surgically approached by thoracotomy through the right lateral sixth intercostal space. The fistula was identified, double ligated and divided. Histopathology confirmed the process to originate from the esophagus and to be patent. The dog was re-examined two weeks and ten months after surgery, with no evidence of recurring clinical signs.

**Conclusions:**

Contrast material swallow study using fluoroscopy was the most reliable diagnostic method. Bronchoscopy may allow the fistula to be visualized, but due to a small fistular opening it can lead to a false negative result. Surgical correction by ligation and dividing of the fistula suggests a good prognosis for early diagnosed and operated TEF.

## Background

An esophageal fistula is an abnormal communication between the esophagus and trachea, bronchus, lung parenchyma or, rarely, the skin [1]. Congenital and acquired tracheoesophageal or bronchoesophageal fistulas in dogs are rarely identified as predisposing factors for recurrent pneumonia [2-6]. To our knowledge, there have been only two previous reports describing tracheoesophageal fistula (TEF) in dogs, one congenital [2] and the other acquired due to an esophageal foreign body [5]. Due to the rarity of the entity, there is very little experience concerning diagnostic methods and surgical correction techniques for TEF in dogs. Therefore, this report aims to contribute to the current literature on suitable diagnostic methods for TEF and to provide follow-up information after successful surgical treatment.

## Case presentation

A seven-month-old, 13.2 kg intact female Spanish Water Dog was presented for further investigation of recurrent respiratory symptoms. Three weeks before referral, the dog had developed an acute onset of lethargy and coughing. Pneumonia was suspected, since chest radiographs showed consolidation of the lungs. Treatment with amoxicillin-clavulanic acid^a^ for ten days resulted in transient improvement in the clinical signs.

On presentation, lethargy, spontaneous cough, tachypnea (respiratory rate 60/min), and moderate crackles on lung auscultation were noted. Coughing was provoked especially after drinking and tracheal palpation. The partial oxygen pressure in the arterial blood (PaO_2_) was 80.5 mmHg. The C-reactive protein (CRP) level was markedly elevated (138 mg/l). Thoracic radiographs revealed alveolar consolidations in the left cranial and left caudal lung lobe, consistent with bronchopneumonia. In abdominal radiographs, the small intestine was gas filled. Supportive treatment and intravenous amoxicillin-clavulanic acid^b^ administration were initiated.

**Figure 1:**
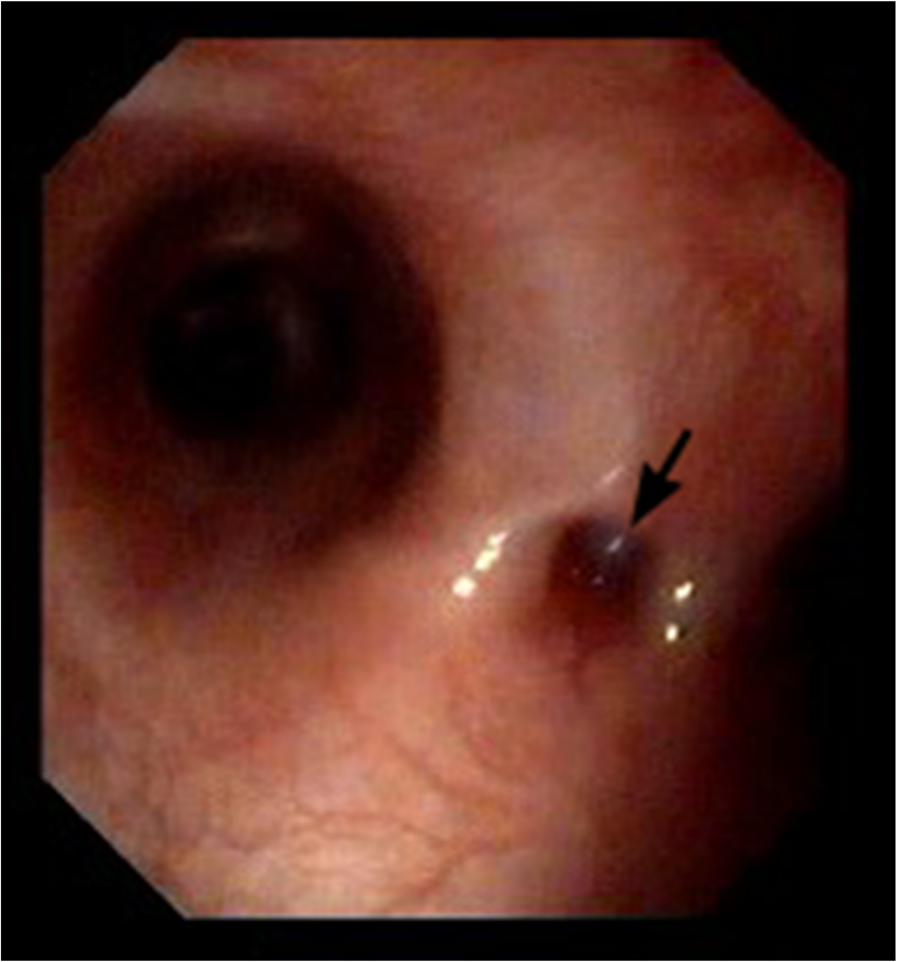
**Endoscopic view of the tracheal bifurcation.** A small hole-like tracheal wall defect is indicated with an arrow.

**Figure 2:**
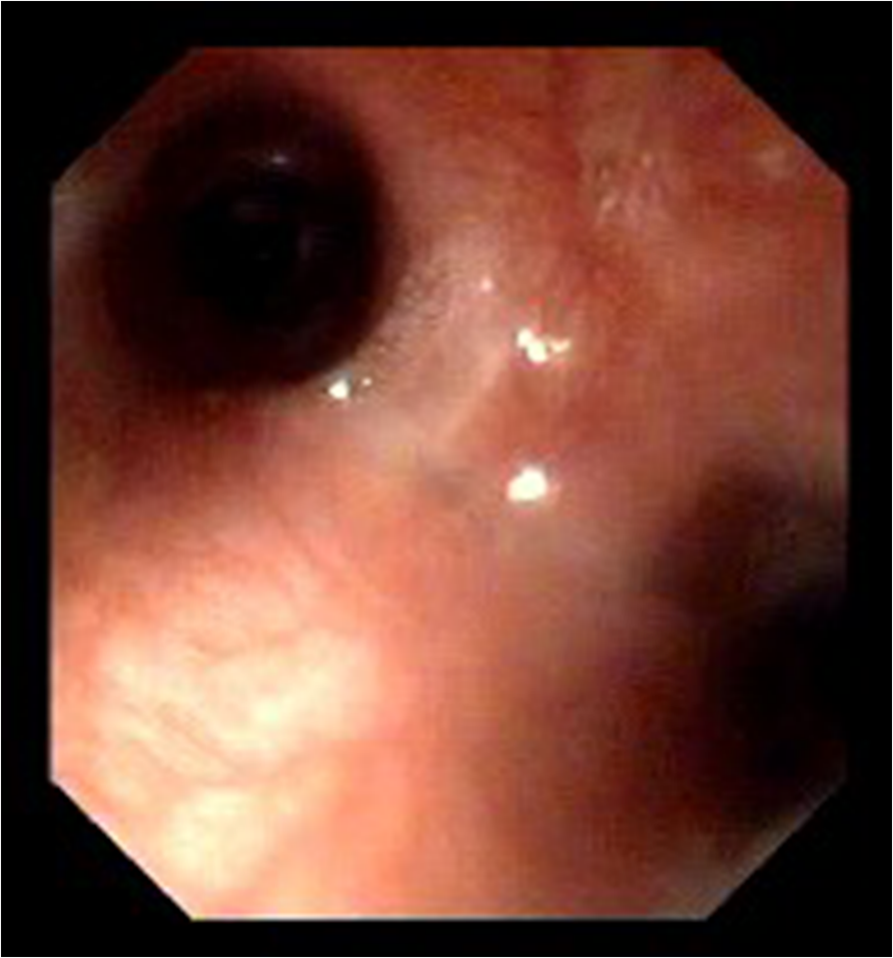
**Endoscopic view of the tracheal bifurcation.** The tracheal wall defect appeared closed after examination of the major bronchi, and as such could easily have been left unrecognized.

The dog was anesthetized and bronchoscopy performed, using a 4.9-mm flexible bronchoscope^c^. At the bifurcation, a small hole-like defect was detected in the tracheal wall (Figure 1). This defect was not visible after examination of the main bronchi (Figure 2). A moderate amount of yellow, viscous, foamy secretion was detected in the bronchial lumen of the left cranial lung lobe. Bronchoalveolar lavage (BAL) was performed on the left cranial lung lobe with a 1-ml/kg bolus of sterile warm (37°C) saline^d^. The cytological examination revealed an elevated total nucleated cell count (1750 cells/μl) and a significantly elevated neutrophil proportion (64.7%). Aerobic and anaerobic cultures were negative, but after enrichment, *Pasteurella stomatis* was isolated in pure culture. The mycoplasma culture isolated *Mycoplasma* spp.

**Figure 3:**
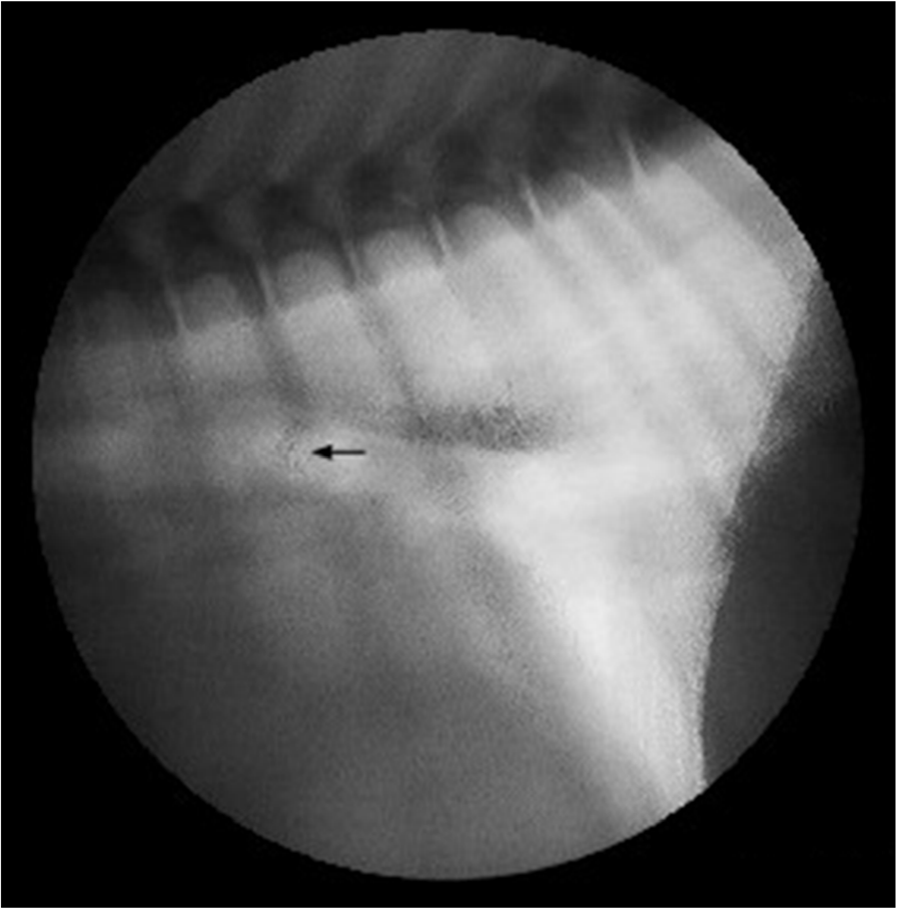
**A contrast material swallow study performed under fluoroscopy with the dog in a standing position.** In a lateral view of the thorax, a small amount of barium contrast medium diluted (1:1) with water was seen to escape ventrally (arrow) from the esophagus along a duct-like structure.

**Figure 4:**
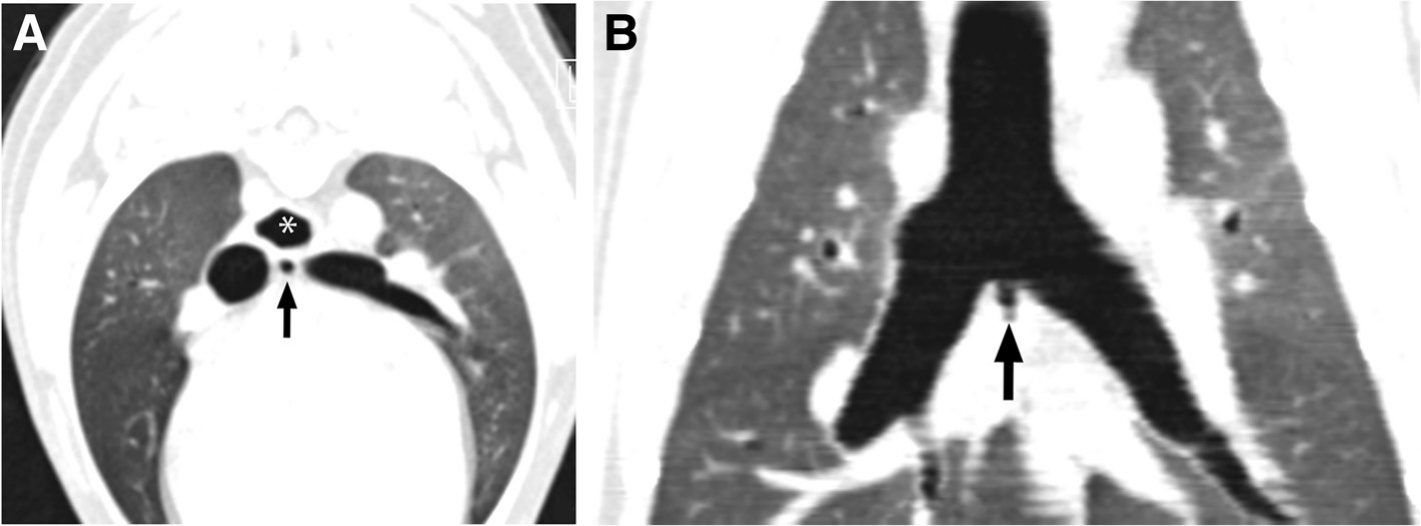
**A thoracic non-contrast CT study of the dog in dorsal recumbency. A)** A transverse image at the level immediately caudal to the carina. The TEF (arrow) is visible between the main bronchi. **B)** A dorsal reconstruction image at the same level as Figure 4**A**. The proximal part of the TEF (arrow) is seen as a pouch between the main bronchi.

To further investigate the defect in the tracheal wall, an upper gastrointestinal contrast material swallow (CS) study was performed with a 10-ml bolus of per orally administered barium contrast medium^e^. A laterolateral thoracic radiograph obtained immediately after the barium swallow showed no leakage of the contrast medium outside the esophagus. Investigations were further continued by a CS study under fluoroscopy with the dog in a standing position. There was no evidence that undiluted barium contrast medium leaked from the esophagus. However, when diluted 1:1 with water, a small amount of contrast medium was seen to escape ventrally from the esophagus, through a duct-like structure (Figure 3). During the procedure, the otherwise normal anatomic structure and function of the esophagus was demonstrated. The thoracic radiograph obtained after CS demonstrated only a small amount of contrast medium in the lower part of the trachea and in the main bronchus. The finding of the CS study under fluoroscopy was indicative of a TEF. To further evaluate the connection between the trachea and esophagus, a computed tomography (CT) scan was performed. In the non-contrast CT, a 6-mm-deep and 3-mm-wide air-filled pouch was seen in the midline in the bifurcation, at the level of the carina, extending caudally between the main bronchi (Figure 4A and B). The finding was consistent with the hole-like structure seen on bronchoscopy. The esophageal part of the TEF was not visible.

**Figure 5:**
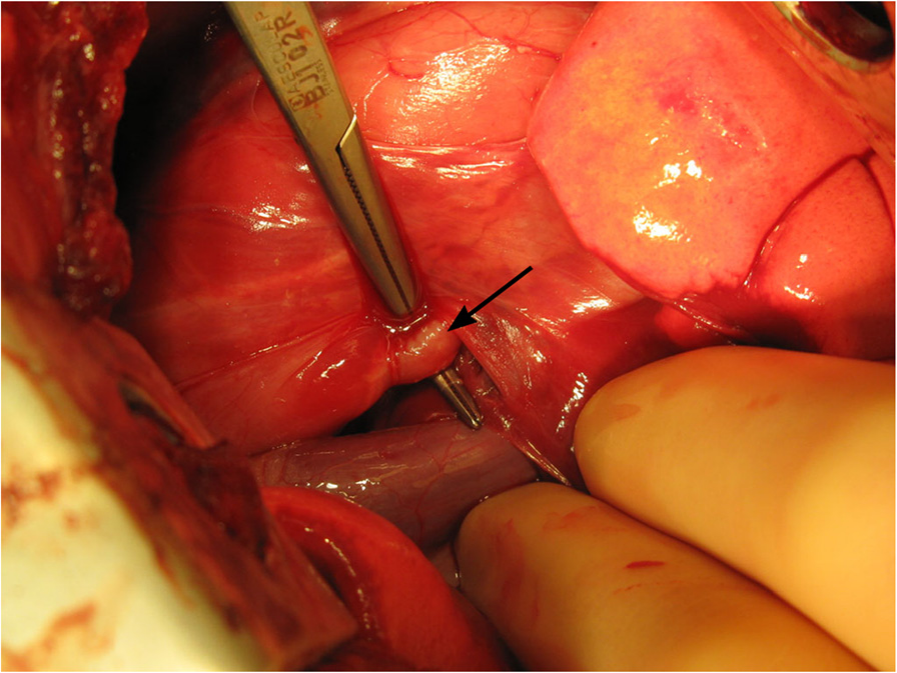
**The TEF was approached by thoracotomy through the right lateral sixth intercostal space.** The fistula (arrow) was double ligated immediately adjacent to the esophagus and approximately 3 cm cranially from the first ligatures. The fistula was completely divided and a tissue sample from the fistula was obtained for histopathology.

Surgical correction of the TEF was scheduled and amoxicillin-clavulanic acid^a^ medication was continued. Prior to surgery, a catheter^f^ was placed along a guide wire^g^ through the TEF from the trachea into the esophagus under endoscopic control. Ventrodorsal and laterolateral thoracic radiographs subsequently obtained showed that the distal opening of the fistula was located at the level of the sixth intercostal space. The TEF was surgically approached by thoracotomy through the right lateral sixth intercostal space. The esophagus and dorsally lying *Nervus vagus* were identified. The depth at which the guide wire was running in the esophageal wall and the exact place where the fistula joined the esophagus could not be identified by palpation. The level of the fistular opening was localized by the light of a 9.8 mm flexible gastroduodenoscope^h^, which was visible through the esophageal wall. The distal end of the fistula (0.5 mm diameter) was bluntly dissected free from the surrounding connective tissue as far cranially as possible (Figure 5). The catheter with guide wire was removed, and the fistula was double ligated with polypropylene 3-0 (Prolene) immediately adjacent to the esophagus. A further double ligature was placed approximately 3 cm cranially from the first ligatures and the fistula was divided. The fistular tissue was fixed in 10% non-buffered formalin and submitted for histopathological examination. The dog recovered well from surgery and was discharged the following day. Oral amoxicillin-clavulanic acid^a^ medication was continued for two weeks. Post-operative analgesia was maintained with fentanyl^i^ and oral carprofen^j^.

**Figure 6:**
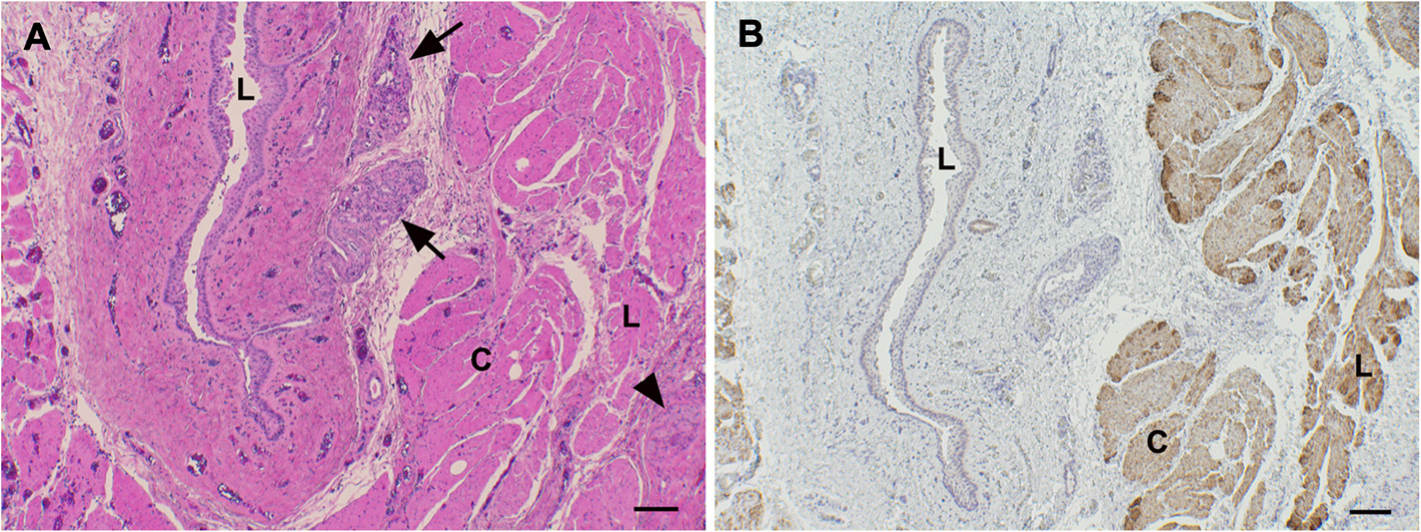
**A histological specimen from the fistula.** The specimen represents a cross section through the mucosa, submucosa and muscularis externa. The slit-like lumen (L) is lined by squamous epithelium. The submucosa contains inactive glandular structures (arrows), and the muscularis externa is comprised of the inner circular (C) and the outer longitudinal (L) layer, and contains plexus structures comprised of unaltered neurons (arrowhead). **A**. Hematoxylin-eosin stained section. **B**. Immunohistology for desmin highlights the striated muscle layers of the muscularis externa and the absence of a lamina muscularis mucosae. Streptavidin peroxidase method, Mayer’s hematoxylin counterstain. Scale bars = 100 μm. (× 50).

The histological examination of cross sections of the excised fistular tissue revealed a tube-like structure with a narrow lumen, lined by stratified squamous epithelium. This was underlain by the lamina propria mucosae, a submucosa containing inactive glandular structures, an inner circular and outer longitudinal skeletal muscle layer (muscularis externa) containing (intermyenteric) plexus structures comprised of several unaltered neurons, and the adventitia (Figure 6).

The dog was re-examined two weeks and ten months after surgery, with no evidence of recurring clinical signs. At the first control visit, the blood leucocyte count, arterial PaO_2_, and CRP were within normal limits, and thoracic radiographs revealed signs of resolving bronchopneumonia. At the second visit, the blood leucocyte count, arterial PaO_2,_ CRP, and thoracic radiographs showed no features suggesting a disease process. In addition, a CS study using fluoroscopy was performed. This did not detect any evidence of leakage of contrast medium from the esophagus. Both the structure and function of the esophagus appeared unaltered.

## Discussion

A congenital TEF is likely to arise as a result of interruption of the events responsible for elongation and separation of the esophageal and tracheal tubes during the fourth fetal week [7]. In the present report, a congenital malformation is considered most likely due to the dog’s young age and the absence of an esophageal foreign body in its history. Histopathology confirms the process to originate from the esophagus and to be patent. The absence of *Lamina muscularis mucosae* in the fistula confirms its origin from the anterior part of the esophagus, since the former is present distally [8]. Furthermore, the lack of any pathological changes in the excised tissue, i.e. absence of a significant inflammatory process and/or abnormal structures, provides strong evidence for its congenital nature [8,9].

This case presented with the classical symptoms encountered in human and canine patients with esophageal fistulas: a chronic cough occurring after drinking due to aspiration of water through the fistula [3,4,10,11], recurrent, chronic lower respiratory tract infections due to continuous aspiration via the fistula [10-13] and gas accumulation in the gastrointestinal tract due to the passage of air from the trachea into the gastrointestinal tract [2,10,11].

The tracheo- and bronchoesophageal fistulas in dogs previously reported were diagnosed with plain thoracic radiographs after barium CS study [2-4,14], indicating that the defect had probably been relatively large in diameter, since a visible quantity of contrast material flowing through it. In the present case, the thoracic radiograph obtained after CS study with diluted contrast medium, demonstrated only a small amount of contrast medium in the lower part of the trachea and in the main bronchus. The presence of contrast material in the airways without visualization of the fistula itself may also be indicative of aspiration and cannot be considered specific for TEF.

In human medicine, numerous diagnostic techniques for TEF have been described, but given the relatively low incidence of TEF; there have been no randomized and controlled trials to support the better use of one method over another. None of the reported diagnostic methods are universally reliable, and the lack of sensitivity makes the diagnosis challenging in mildly affected patients [13,15-19]. The initial radiological investigation consists of plain thoracic and abdominal radiographs, which may raise suspicion of TEF by showing evidence of aspiration and gastric dilatation [2,12,13]. The most frequently described radiological diagnostic techniques to confirm the presence of TEF are CS study and the tube esophagogram [13,15,19], where the contrast medium is injected into the esophagus under pressure and observed via plain radiographs and fluoroscopy.

Inconstant patency of TEF is associated in a dog with a slit-like opening of the esophageal fistula orifice [2]. In humans, an esophageal mucosa fold occluding the esophageal side of the fistula, or an oblique course of the fistula to the esophagus has been described, which might cause the esophageal mucosa adjacent to the orifice to function as a valve during swallowing, compressing the anterior esophageal wall against the fistula and occluding its lumen [13,20]. Additionally, a small diameter of the orifice, as in our case, can complicate the diagnosis of TEF. Diluted contrast medium may pass more easily through fistulas with a narrow lumen [12,14], thereby rendering a diagnosis possible in suspected cases where CS study with undiluted contrast material is negative. It should be noted that, as in our case, diluted contrast medium may be more readily observed via fluoroscopy than plain thoracic radiographs.

The use of CT has been increasing in recent years. According to previous studies [12,16,18,21], contrary opinions on the use of CT in humans exist. A recent study by Mahalik et al. [18] stated that a CT scan could provide good anatomic delineation, but might not help in surgical decision making. To the authors’ knowledge, the present case is the first reported study in which CT is used in a dog in an attempt to identify TEF. Non-contrast CT is not helpful in the diagnosis, since it does not enable the visualization of the distal esophageal part of the TEF. However, the air-filled pouch between the main bronchi would raise a suspicion of abnormal anatomy, even if the bronchoscopy does not reveal the orifice of the TEF. In neonate children, the concurrent use of air during CT has been a valuable aid in the diagnosis of TEF, serving as a negative contrast medium [21,22].

Endoscopic techniques, such as bronchoscopy and esophagoscopy, are complimentary techniques for diagnosing TEF. They may allow the fistula to be directly visualized and its origin to be exactly localized [10,13]. Nevertheless, endoscopic techniques can lead to a false negative result, and repeated examinations may be necessary, as the fistula can be difficult to identify in a single attempt [13]. In our case, the tracheal wall defect was diagnosed during bronchoscopy. The defect was clearly visible only in the early stage of bronchoscopy, and when later reassessed appeared closed, and as such could easily had been left unrecognized. A reason for poor identification of the fistula could be the small size of the orifice, which might easily collapse due to pressure changes during ventilation. This indicates that a negative diagnosis in endoscopy is not exclusive and other diagnostic methods are needed when TEF is suspected.

In dogs, congenital or acquired bronchoesophageal fistulas are mainly seen in association with an esophageal diverticulum and foreign body [14]. In humans, esophageal atresia is associated with congenital TEF in the vast majority of patients (85–88%) [23,24]. In our case, the anatomic structure and function of the esophagus were unaltered both prior to and after surgery. This rules out any relevant concomitant anomalies and is considered a good prognostic indicator for surgical correction.

Surgery consists of ligation and division of the fistula and, if needed, repair of the tracheal and esophageal walls [10,25]. In the present case, the connection between the trachea and esophagus was identified with a catheter, as previously described [10,24]. Thoracotomy was performed in the sixth intercostal space according to guide wire location in thorax radiographs. The proximal end of the fistula was not further searched for due to the moderate length of the fistula, challenging location between important structures, thick connective tissue, and adhesions surrounding the fistula. In humans, surgical repair of the proximally located fistula is approached through the cervical route along the anterior border of the sternocleidomastoid muscle. An approach via thoracotomy is taken when the fistula is located more distally, at the level of the carina, or when partial pulmonary resection is required at the same time [10,20]. TEFs of newborns have also been successfully repaired thoracoscopically [26].

Due to the low incidence of reported TEFs in dogs and the lack of long-term follow-up data, no information on complications or the recurrence rate is available. In our case report, the dog’s postoperative course was uneventful, with no signs of recurrence within the ten-month follow-up period. Recurrent TEF is one complication of surgical therapy among children with congenital esophageal atresia and TEF [23]. TEF recurs in less than 5% to 8% of cases, most often 2 to 18 months after initial repair [24,25]. Ligation of the fistula rather than complete division increases the incidence of recurrent TEF [25].

## Conclusions

Recurrent respiratory infections in young dogs require a thorough examination to identify possible predisposing pathological conditions. TEF needs to be considered as a possible cause for repeated respiratory tract infections. CS study using fluoroscopy was the most reliable diagnostic method in our case. Diluted contrast material is advisable to use in suspected cases where CS study with undiluted contrast material is negative. Bronchoscopy may allow the fistula to be visualized, but due to a small fistular opening it can lead to a false negative result. When non-contrast CT is not helpful in the TEF diagnosis, air in the esophagus as a negative contrast medium could be more informative. In the case described, surgical correction by ligation and dividing of the fistula remained successful after ten months, and suggests a good prognosis for early diagnosed and operated TEF.

## Endnotes

^a^Synulox, Pfizer Oy Animal Health, Helsinki, Finland.

^b^AmoxClav, Hexal AG, Holzkirchen, Germany.

^c^Olympus GIF N180, Olympus Medical Systems Europa GMBH, Hamburg, Germany.

^d^Sodium Chloride 0.9% Injections USP, B. Braun Medical Oy, Helsinki, Finland.

^e^Mixobar colon, 1.0 mg/ml, E-Z-EM, Anjou, Canada.

^f^Buster Sterile Dog Catheter 1.3 × 500 mm, 4 FG, Kruuse, Langeskov, Denmark.

^g^0.025′ guidewire VisiGlide, 2700 mm, Olympus, Hamburg, Germany.

^h^Pentax EG2940K, Pentax GMB, Hamburg, Germany.

^i^Durogesic, Janssen-Cilag Oy, Espoo, Finland.

^j^Rimadyl, Pfizer Oy Animal Health, Helsinki, Finland.

## Abbreviations

TEF: Tracheoesophageal fistula; PaO2: Partial oxygen pressure in the arterial blood; CRP: C-reactive protein; BAL: Bronchoalveolar lavage; CS: Contrast material swallow; CT: Computed tomography.

## Competing interests

None of the authors has any financial or personal relationships that could inappropriately influence or bias the content of this paper.

## Authors’ contributions

Clinical examination, diagnosis and treatment of animal were performed by PSK, SJV, MMR and OMLV. AKL performed the analysis and interpretation of the CT findings. AK performed the analysis and interpretation of the histological findings. All the authors helped to draft the manuscript. All authors read and approved the final manuscript.
